# Repeated Caesarian Section

**Published:** 1914-05

**Authors:** 


**Repeated Caesarian Section.** Asa B. Davis, N. Y., Cleveland Med. Jour., Feb. 1914. The N. Y. Lying-in Hospital records 495 Caesarian Sections since 1893. The total maternal mortality was 50. Repeated operations have been done 69 times. Rupture through the uterine scar was noted in 6 subsequent labors.

